# Changing handedness after nerve reconstruction in brachial plexus birth palsy

**DOI:** 10.3389/fneur.2023.1284945

**Published:** 2024-01-08

**Authors:** Chenglun Yao, Jie Song, Jiayu Sun, Weijun Tang, Liang Chen, Yudong Gu

**Affiliations:** ^1^Department of Hand Surgery, Huashan Hospital and Institutes of Biomedical Sciences, Fudan University, Shanghai, China; ^2^NHC Key Laboratory of Hand Reconstruction (Fudan University), Shanghai, China; ^3^Shanghai Key Laboratory of Peripheral Nerve and Microsurgery, Shanghai, China; ^4^Institute of Hand Surgery, Fudan University, Shanghai, China; ^5^Department of Radiology, Huashan Hospital, Fudan University, Shanghai, China

**Keywords:** brain function lateralization, handedness switching, handedness-speech center, obstetric brachial plexus injury, postoperative rehabilitation

## Abstract

**Purpose:**

Right obstetric brachial plexus injuries (OBPI) often lead to left-handedness before limb function is restored post-surgery. A pertinent question arises about promoting a transition from left to right-handedness. We hypothesized that, with the decrease in neuroplasticity, handedness switching is not only difficult, but also reduces handedness-speech lateralization, impaired motor adaptability, and compromised language proficiency.

**Methods:**

We retrospectively analyzed clinical data from January 1996 to January 2012 at our hospital. Participants were divided into intervention or control groups based on handedness switching. We compared handedness and computed lateral quotient (LQ) and lateralization index (LI) for handedness-speech center. Additionally, we assessed dominant hand’s writing speed, language function, and IQ. Associations between absolute LI and LQ values, writing speed, language scores, and IQ were examined.

**Results:**

Nineteen extended Erb’s palsy participants were enrolled, eight in the intervention group, and 11 in the control. No right-handed individuals were found in either cohort. The intervention group had significantly lower LQ and LI values, and fewer achieved normal writing speed. Yet, no notable disparities in language scores or IQ emerged. Notably, we established correlations between motor finesse, handedness degree, and handedness-speech lateralization.

**Conclusion:**

For right extended Erb’s palsy, shifting handedness is nearly unfeasible, and such an endeavor could trigger a reduction in handedness-speech lateralization magnitude and diminished motor finesse.

## Introduction

Obstetric brachial plexus injuries (OBPI) ensues from forceful head and shoulder separation during labor, provoking traction injury. Upper OBPI includes Erb’s palsy (C5-C6) and extended Erb’s palsy (C5-C6-C7). Unlike Erb’s palsy, extended Erb’s palsy affects C7, inflicting heightened damage to C5 and C6 (1). Consequently, the probability of necessitating nerve reconstruction surgery notably surges in the latter. Although nerve reconstruction yields positive outcomes, the recuperation of limb function extends over 3–4 years (2). In this duration, patients often manifest a penchant for employing their left hand following right-sided injuries. Hence, the quandary of whether to switch handedness from left to right emerges as a conundrum.

Brain function lateralization refers to the asymmetry in cerebral cortex activation. It is a phenomenon commonly observed in vertebrates and is known to enhance brain efficiency. For example, pigeons with lateralized vision find more grain particles scattered in pebbles (3); chimpanzees catch more termites using detection tools with dominant hands (4). In humans, the most prevalent form of lateralization is handedness, with 90% of the population being right-handers. The center responsible for handedness is typically located in the left hemisphere of the brain. Interestingly, handedness can show early indications, such as the preference for thumb sucking from 15 weeks of gestation (5). Another significant aspect is the speech center, which comprises the motor, auditory, writing, and visual functions of language. Speech center is preliminarily established during the fetal stage and, similar to handedness, is usually located in the left hemisphere (6). Studies demonstrated that the lateralization of handedness and speech dominance tends to occur in the same hemisphere (7). This may be attributed to the efficiency promotion of speech expression through hand gestures and body language (8, 9). Lateralization patterns can be influenced by various factors, including central or peripheral nerve injuries (10, 11). For instance, Woods’ Wada test on 237 patients revealed that structural injuries in the speech area, speech lateralization reshaped and altered handedness (12). Conversely, handedness switching after OBPI could influence speech lateralization (7). Moreover, the degree of lateralization has been found to be closely related to task performance. Higher degrees of handedness are associated with stronger fine movement abilities (13); individuals with stronger speech lateralization have higher IQs and better reading comprehension skills (14).

Based on these observations, we hypothesized that as neuroplasticity decreases with age, attempting to switch handedness back for patients who have developed left-handedness after surgery may not only be challenging but also lead to a reduction in the degree of handedness-speech lateralization, weakening motor dexterity, and language performance. To verify this hypothesis, handedness assessment, task-related fMRI, evaluation of dominant hand’s writing speed, language function assessment, and IQ testing were employed.

## Subjects and methods

### Subjects

Ethics approval (Program number: KY2020-1219) was obtained from our hospital’s Ethics Committee. Clinical data were retrospectively collected from right upper obstetric brachial plexus injury (OBPI) patients meeting brachial plexus reconstruction criteria, treated between January 1996 and January 2012 at our center. Enrolled subjects or their parents provided informed consent.

Inclusion criteria: (1) Diagnosed with right Erb’s or extended Erb’s palsy, underwent brachial plexus nerve reconstruction at our center. (2) ≥4 years follow-up, evaluated using Gilbert/Raimondi assessment (MRC grade) (15). “Basically normal” recovery: shoulder ≥4, elbow ≥4, and hand function ≥5. (3) Age ≥ 7 years. (4) Understanding study and giving informed consent. Exclusion criteria: (1) Conditions affecting results (e.g., cerebral hemorrhage). (2) Mental conditions hindering cooperation. (3) Uncorrectable visual acuity (< 0.8). (4) Recent/pending clinical trial participation. (5) Inability to tolerate/exclude participation.

### Grouping

Participants implementing handedness switching included parents, rehabilitators, teachers, and willing patients. Training incorporated daily tasks like writing, painting, grooming, and dining. “Intervention” required ≥3 years of training; shorter periods were “control.”

### Assessment of handedness

The Edinburgh Handedness Inventory (16) was used, modified to accommodate Chinese traditions. LQ was calculated as (L − R)/(L + R) × 100 (L: left-hand activities, R: right-hand activities). LQ ≤ −50 indicated left-handedness, LQ ≥ +50 indicated right-handedness, −50 < LQ < +50 indicated bilateral handedness. Greater LQ absolute value indicated stronger lateralization (17).

### Evaluation of handedness-speech lateralization

Semantic association task fMRI was employed to identify handedness-speech center activation voxels. After semantic association, response time and judgment accuracy were calculated to deem that the activation maps of semantic association could accurately reflect handedness-speech center lateralization (18).

Magnetic resonance imaging was performed using the GE Discovery MR 750 3.0 T United States general superconducting MR scanner with an eight-channel head coil. The stimulation task was conducted using the SA-9939 brain functional visual and auditory stimulation system and the button feedback system in the examination room (Shenzhen Meide Medical).

Language task-related fMRI was conducted using the following steps:

A T1WI cross-sectional image scan was performed with the following parameters: Repeat time (TR) = 2,000 ms; echo time (TE) = 3.2 ms; Turning Angle (FA) = 12°; field of view (FOV) = 24 cm × 24 cm; layer thickness = 4 mm; interval = 1.2 mm; matrix: 192 × 256; and voxel size (V) = 0.7 mm. Blood Oxygenation Level Dependent (BOLD) fMRI was performed with the following parameters: TR = 2,000 ms; TE = 30 ms; FA = 90°; FOV = 24 cm × 24 cm; scanning layers = 35, with each layer containing 240 frames of functional images; V = 2.0 mm; matrix: 128 × 128. The layer thickness and interval were the same as the SE sequence.

#### Semantic association task

The stimulus program presented theme words on the monitor in the examination room. The subject silently associated with the theme word for 10 s, followed by a 10-s rest period. This process was repeated nine times, with the theme word changing each time.

#### Semantic judgment task

Similar to the semantic association task, the subject was presented with pairs of words and pictures and had to make a “consistent” or “inconsistent” judgment by pressing a button. The graphic stimulation lasted for 6 s, followed by a 6-s rest period. This process was repeated 15 times, with the stimulus changing each time.

#### Data processing and analysis

Data underwent preprocessing in SPM12 on Matlab R2019a. This included head motion correction, brain normalization, and image smoothing. Statistical models, functional matrices, and activation thresholds were employed to locate activation voxels in relevant brain areas.

#### Semantic association fMRI reliability judgment criteria

Response time and judgment accuracy from semantic judgment fMRI were used. If the maximum response time of the subject differed by less than three standard deviations from the whole group, with a judgment accuracy greater than 85%, and the area of the handedness-speech center was activated, the activation voxel obtained by the semantic association fMRI was considered suitable for further analysis (19). This fMRI operation and analysis were conducted by radiology professionals at our hospital.

#### LI calculation for handedness-speech lateralization

The LI was calculated as follows: (activated voxels in the left cortex − activated voxels in the right cortex)/activated voxels in the bilateral cortex (20). The well-recognized standard was: LI > +0.20 indicated left lateralization; LI < −0.20 indicated right lateralization; and no significant lateralization was between −0.20 and +0.20 (21). A higher absolute value of LI reflects a higher degree of lateralization of the handedness-speech center (22).

### Dexterity of the dominant writing hand

The writing speed was used as an indicator of the dexterity of the dominant hand in performing fine movements (13). If the subject was ambidextrous, the writing speed was measured using the most commonly used writing hand, which is the dominant writing hand. Results were excluded if the subject’s handedness side and dominant writing hand were inconsistent. Luria’s method (23) was employed, and a normal writing speed should be no less than 39.4 words per minute (24). The difference in the proportion of subjects who reached this writing speed value between the two groups was compared.

### Language function

Language function was assessed using the Aphasia Battery of Chinese, which was modified from The Western Aphasia Battery (25).

### IQ

The IQ questionnaire was utilized to test the subject’s intelligence (14, 26).

### Statistical methods

Data were presented as mean ± standard deviation (M ± SD). Chi-square or Fisher’s exact test compared handedness distribution and normal dominant hand’s writing speed proportions. Using independent *t*-test for normal distribution and Mann–Whitney U test for skewed distribution, LI values, LQ values, language scores, and IQ were compared between groups. Linear regression analyzed LI-LQ correlation, writing speed, language score, and IQ. Statistical software was used for data processing, with significance at *p* < 0.05.

## Results

Nineteen subjects with extended Erb’s palsy were included, comprising 11 males and eight females, with ages ranging from 7 to 26 years and a median age of 9 years. Eight subjects were assigned to the intervention group, while 11 were assigned to the control group. Table 1 presents the demographic data, function assessment, surgery timepoint, and reconstruction method of the subjects.

**Table 1:** Demographic data, function assessment, surgery timepoint, and reconstruction methods (*n* = 19).

No.	Sex	Age	Assessment			Timepoint (month)	Reconstruction
			Shoulder	Elbow	Hand		
Intervention group							
1	F	11	5	5	5	4	C5 → PC
							C6 → LC
							AN → SN
2	M	13	5	5	5	4	C5 → MT + SN
							C6 → ADUT + PDUT
3	M	10	5	5	5	4	C5 → ADUT + PDUT
							C6 → PDUT + MT
							AN → SN
4	M	12	4	5	5	5	C5 → PDUT
							C6 → ADUT
							AN → SN
5	F	7	5	5	5	5.5	C5 → ADUT + PDUT
							C6 → PDUT + MT
							AN → SN
6	F	9	5	5	5	7	C5 → ADUT + SN
							C6 → MT + PDUT
7	M	26	4	5	5	4	C5 → PDUT
							C6 → ADUT
							AN → SN
8	M	12	5	5	5	5	C5 → PDUT
							C6 → ADUT
							AN → SN
Control group							
9	F	8	4	5	5	3.5	AN → SN
							IN (7-8-9) → MN
10	F	9	5	5	5	6	AN → SN
							IN (7–8) → MN
11	M	7	5	5	5	4	C5 → ADUT + SN
							C6 → PDUT + MT
12	M	11	5	5	5	5	C5 → PDUT + MT
							C6 → ADUT + PDUT
							AN → SN
13	M	7	5	5	5	4	C5 → PDUT + MT
							C6 → ADUT + PDUT + SN
14	M	7	5	5	5	5	C5 → ADUT + SN
							C6 → PDUT + MT
15	M	13	5	5	5	3.5	C5 → ADUT + PDUT
							AN → SN
16	F	8	5	5	5	4	C5 → PDUT
							AN → SN
							IN (7-8-9) → MN
17	F	11	5	5	5	5	C5 → PDUT + PDMT
							C6 → ADUT + PDUT
18	M	7	5	5	5	5.5	C5 → ADUT + SN
							C6 → MT + PDUT
19	F	8	5	5	5	4	C5 → MT
							AN → SN
							IN (7-8-9) → MN

PC, Posterior cord; LC, Lateral cord; AN, Accessory nerve; SN, Suprascapular nerve; MT, Middle trunk; ADUT, Anterior division of upper trunk; PDUT, Posterior division of upper trunk; IN, Intercostal nerves; MN, Musculocutaneous nerve; and PDMT, Posterior division of middle trunk.

### No right-hander achieved; intervention group had lower degree of handedness

Handedness assessment (Supplementary Table S2) indicated the intervention group had varied hand preference, while controls leaned left-handed. In the intervention group, seven were ambidextrous, one left-handed; controls were all left-handed (*p* = 0.0002). Average LQ absolute value was lower in intervention (38.9 ± 20.4) than controls (91.3 ± 13.5, *U* = 1.5, *p* = 0.0004).

### The degree of handedness-speech lateralization was lower in intervention group

#### Semantic judgment determined the accuracy of semantic associations

The semantic judgment task indicated that the maximum response time was 901.7 ms (< 3 SD from avg. 843 ± 101 ms), and the judgment accuracy was 89.2% (ranging from 86.7 to 100%) in the intervention group. In the control group, the figures were 877.1 ms (< 3 SD from 821 ± 93 ms) and 89.7% (ranging from 86.7 to 100%; Supplementary Table S3). The activation regions of semantic judgment encompassed the areas where the handedness-speech center was activated during semantic association in both groups.

#### Intervention group had lower absolute value of LI

**Figure 1:**
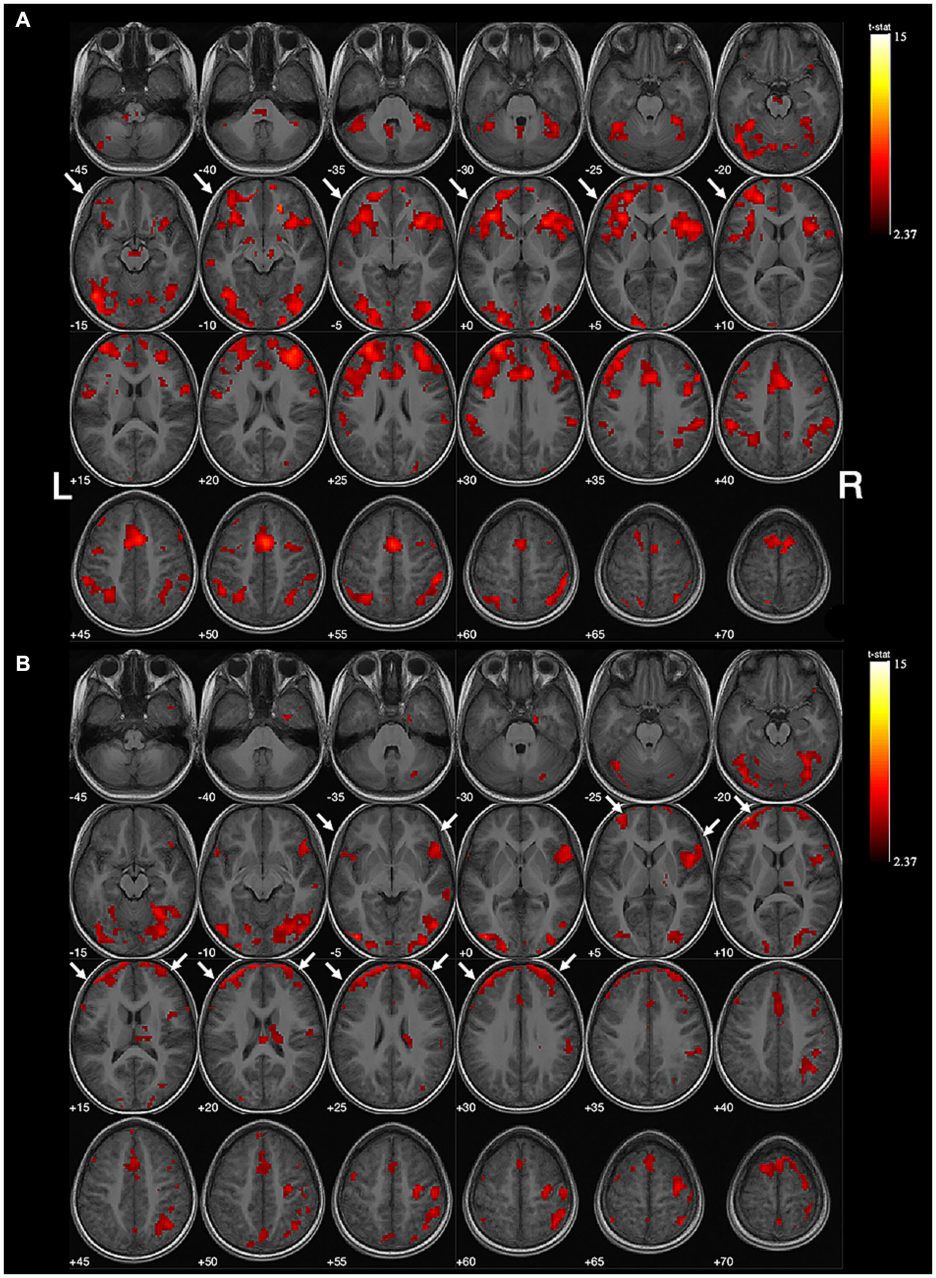
Example of no obvious lateralization of handedness-speech. Subject No. 6: **(A)** The activation map of semantic judgment contains the regions of handedness-speech center in semantic association (white arrows). **(B)** The semantic association images showed more activated voxels on the left hemisphere (white arrows), LI = +0.18, indicating no significant handedness-speech lateralization. Activation maps obtained from task-related fMRI were overlaid onto the standard anatomical brain in radiological position. Color bars are shown on the right side, marked with the range of normalized correlation coefficient values associated with the activations (threshold = 2.37).

**Figure 2:**
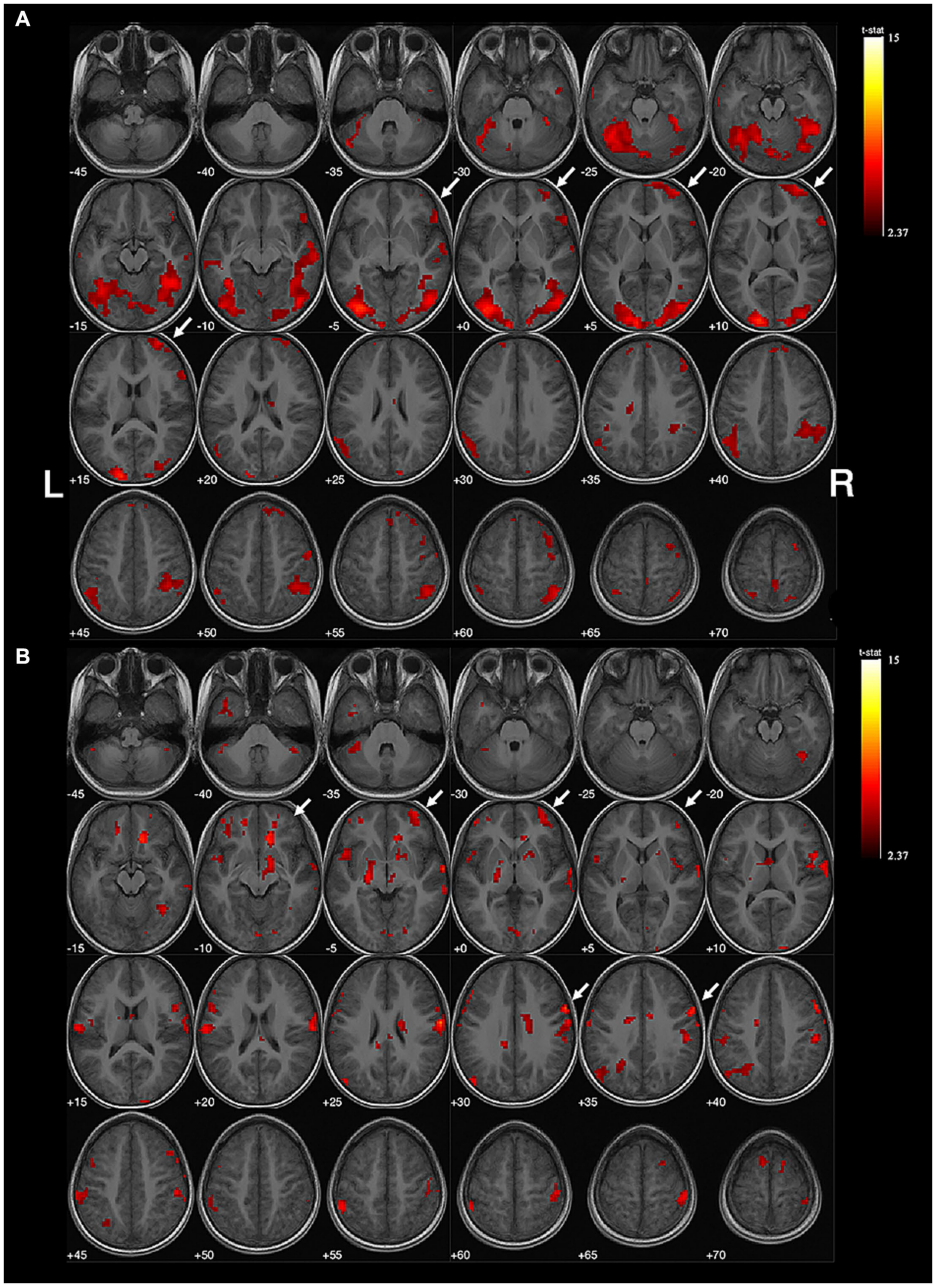
Example of right-sided handedness-speech lateralization. Subject No. 12: **(A)** The activation map of semantic judgment contains the regions of handedness-speech center in semantic association (white arrows). **(B)** The semantic association images showed more activated voxels on the right hemisphere (white arrows), LI = −0.41, indicating right-sided handedness-speech lateralization. Activation maps obtained from task-related fMRI were overlaid onto the standard anatomical brain in radiological position. Color bars are shown on the right side, marked with the range of normalized correlation coefficient values associated with the activations (threshold = 2.37).

The mean activated voxels in the intervention group were 7,365 ± 4,189 in the left hemisphere and 7,379 ± 3,235 in the right, resulting in an average absolute value of LI of 0.25 ± 0.18. In the control group, the 11 subjects had an average of 2,604 ± 1,049 activated voxels in the left hemisphere and 10,062 ± 3,468 in the right, with an average absolute value of LI of 0.56 ± 0.17 (Supplementary Table S3). The absolute value of LI was significantly lower in the intervention group (*U* = 10.00, *p* = 0.0057). Figures 1, 2 depict two examples of the fMRI images.

### Intervention group had lower proportion of normal writing speed

In the intervention group, all subjects exhibited a dominant writing hand on the right side, with seven being ambidextrous and one being left-handed (result excluded). Among them, three subjects achieved normal writing speed (Supplementary Table S3). In contrast, in the control group, consistent with handedness, all subjects showed left dominance for writing, with nine out of 11 subjects reaching normal writing speed (Supplementary Table S3). Fewer subjects in the intervention group reached normal writing speed (*χ^2^* = 4.923, *p* = 0.0265).

### No significant difference in language score or IQ

The average language score was 68.4 ± 1.8 in the intervention group and 69.4 ± 1.4 in the control group, and there was no statistically significant difference between them (*t* = 1.380, *p* = 0.1855). Similarly, the average IQ was 99.3 ± 6.7 in the intervention group and 94.5 ± 5.0 in the control group, and no significant difference was observed (*t* = 1.692, *p* = 0.1112).

### The absolute value of LI was positively correlated with the absolute value of LQ and dominant hand’s writing speed, but not related to language score or IQ

**Figure 3:**
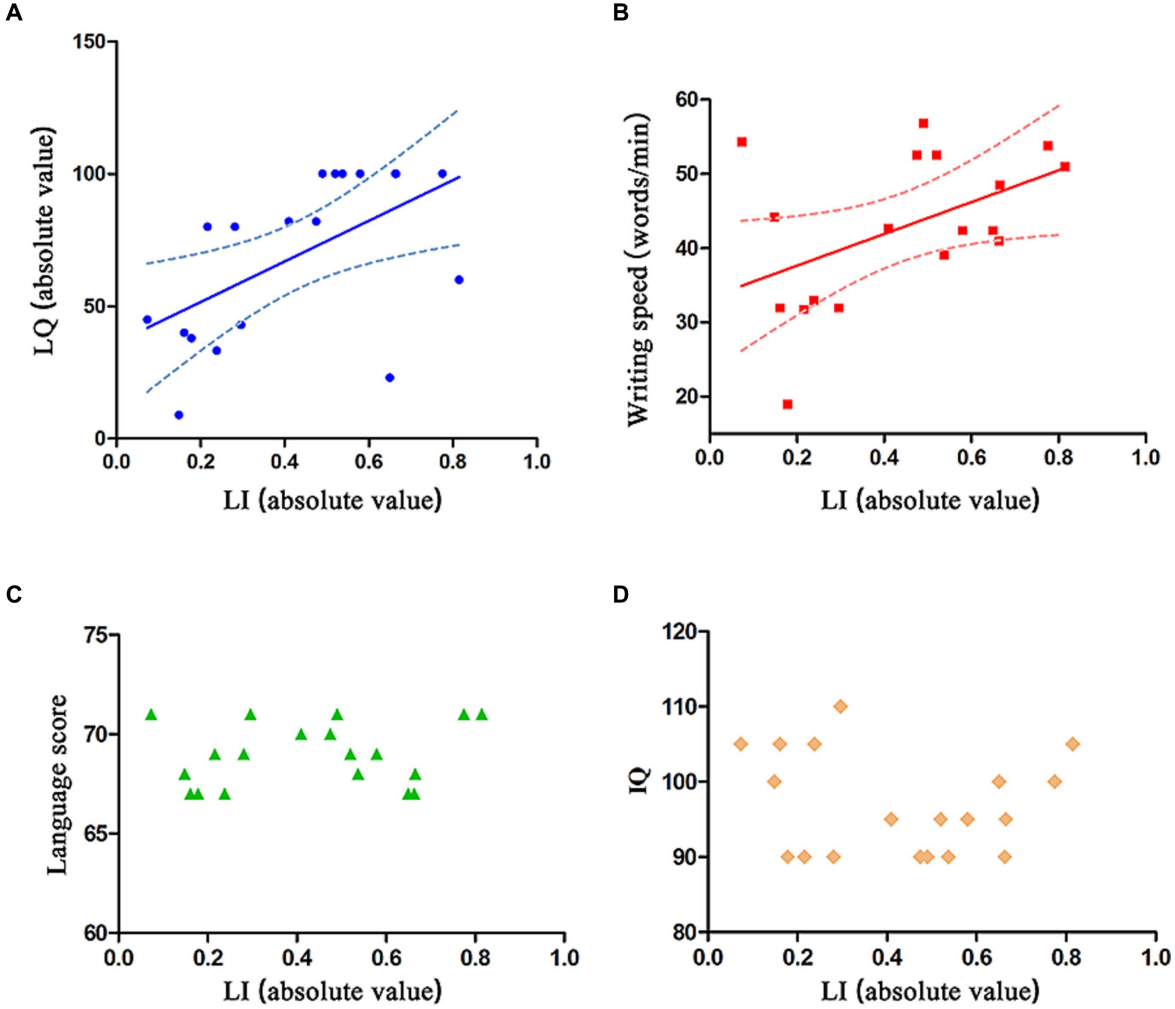
Relation between absolute value of LI and absolute value of LQ, dominant hand’s writing speed, language score, and IQ. **(A,B)** The absolute value of LI is positively correlated with the absolute value of LQ and dominant hand’s writing speed. The solid line represents the fitting line, and the dashed line represents the 95% confidence interval. **(C,D)** The absolute value of LI is not correlated with language score or IQ.

The absolute value of LI was found to have a positive correlation with the absolute value of LQ (*p* = 0.0121, *β* = 0.56, *R*^2^ = 0.32, 95% CI = −82.1 ~ 29.1, Figure 3A) and with the dominant hand’s writing speed (*p* = 0.0410, *β* = 0.49, *R*^2^ = 0.24, 95% CI = −23.3 ~ 43.4, Figure 3B). However, there was no correlation between the absolute value of LI and language score (*p* = 0.4951, *R*^2^ = 0.02780; Figure 3C) or IQ (*p* = 0.5461, *R*^2^ = 0.02183; Figure 3D).

## Discussion

This study was to address a common clinical question posed by right upper OBPI patients and their parents after nerve reconstruction, namely, whether it is necessary to convert handedness to restore right-handedness. The 19 extended Erb’s palsy patients were divided into an intervention group (eight subjects) and a control group (11 subjects). Various aspects, including handedness status, brain lateralization, flexibility of the dominant hand, language function, and IQ, were evaluated and compared. The results revealed that none of the patients in the intervention group successfully completed the switch to right-handedness. fMRI demonstrated that handedness switching training reduced the degree of handedness-speech lateralization. Additionally, the proportion of subjects who achieved normal writing speed in the intervention group was lower than that in the control group. However, there were no significant differences in language function or IQ between the two groups. Correlation analysis further showed that the degree of handedness-speech lateralization was positively correlated with the degree of handedness lateralization and dominant hand’s writing speed. These findings suggest that handedness switching after right extended Erb’s palsy is nearly impossible to achieve, as it does not lead to a complete change from left to right handedness. Moreover, the decrease in the degree of handedness-speech lateralization may result in side effects such as reduced hand flexibility.

The handedness center and the speech center are often located in the same hemisphere. Remodeling of lateralization in one center may cause synchronous conversion in the other. Therefore, the two centers are collectively referred to as handedness-speech center lateralization (27). The lateralization of the handedness-speech center begins to develop in the fetal stage (6) and is generally established by the age of 2 (28). Most of the population are right-handed, which means the handedness-speech center is usually left-lateralized. In the control group of this study, all 11 subjects were left-handed, and their handedness-speech center was lateralized on the right hemisphere, indicating that they used the left hand predominantly due to dysfunction of the right upper limb, leading to a shift in handedness-speech lateralization from left to right. However, in the intervention group, after more than 3 years of handedness switching, most of the eight subjects became ambidextrous, but not right-handed, and their handedness-speech center showed no lateralization tendency. These results suggest that handedness switching training can cause a shift in lateralization of the handedness-speech center from right to left to some extent. However, this form of shift is no longer sufficient to fully change the lateralization side after the right upper extremity function is essentially restored (at least 4 years) in brachial plexus reconstruction patients. It is speculated that although children generally have strong neural plasticity, the lateralization of language development in children after the age of 2 has become stable, making it difficult to synchronously transfer handedness-speech lateralization as patients grow older. However, the exact mechanism behind this phenomenon requires further investigation.

Writing necessitates precise coordination of upper extremity joints, with vital roles of elbow, wrist, and hand joint synergy for writing speed improvement (29). Therefore, the dominant hand’s writing speed serves as an indicator of dexterity in completing fine movements (13). In the study, seven ambidextrous intervention subjects, switching to right-handed writing, saw only three achieving normal speed, lower than nine of 11 controls. Since previous studies have demonstrated that a higher degree of handedness-speech lateralization correlates with greater flexibility of fine motor skills (30), the reduced flexibility observed in the intervention group is evidently related to the non-lateralization of the handedness-speech center (Figure 3B). This suggests that handedness switching training for these patients not only fails to restore right-handedness but also leads to a decrease in hand dexterity. Literature has shown a positive correlation between the degree of handedness-speech lateralization and reading ability (31) and IQ (14). However, this study did not reveal any significant correlation between the degree of handedness-speech lateralization and these two functional indicators (Figures 3C,D). Definitive conclusions may require larger samples and longer studies.

### Conclusion

In cases of right extended Erb’s palsy, after upper limb function is restored through nerve reconstruction, handedness switching is almost impossible to reverse the established left-handedness or convert the lateralization of the handedness-speech center. However, the resulting decrease in the degree of handedness-speech lateralization may negatively affect the flexibility of fine movements.

### Limitations

This pioneering research explores handedness switching training post nerve reconstruction in right upper OBPI, with innovative significance. Limitations include: (1) No observation of multi-stage growth, hindering dynamic handedness-speech assessment. (2) Potential natural left-handers, affecting results. (3) Predominantly juveniles, unclear adult applicability. Hence, the possible impact of these deficiencies on the results should be taken into account when interpreting the clinical significance of this study.

## Data availability statement

The original contributions presented in the study are included in the article/Supplementary material; further inquiries can be directed to the corresponding author.

## Ethics statement

The studies involving humans were approved by Huashan Hospital’s Ethics Committee. The studies were conducted in accordance with the local legislation and institutional requirements. Written informed consent for participation in this study was provided by the participants’ legal guardians/next of kin.

## Author contributions

CY: Conceptualization, Data curation, Formal analysis, Resources, Writing – original draft, Writing – review & editing, Methodology. JSo: Conceptualization, Writing – review & editing, Methodology, Resources, Writing – original draft. JSu: Writing – review & editing. WT: Formal analysis, Software, Writing – review & editing. LC: Conceptualization, Funding acquisition, Supervision, Writing – review & editing. YG: Supervision, Writing – review & editing, Funding acquisition.
