# Supplementary material for: Changing handedness after nerve reconstruction in brachial plexus birth palsy
Source: Front Neurol. 2024 Jan 8;14:1284945. doi: 10.3389/fneur.2023.1284945 (PMC10800742; doi:10.3389/fneur.2023.1284945)
Supplement: Supplementary file 1 [file Table_1.doc]

| **Table S1. Handedness Assessment (n = 19)** | | | | | | | | | | | | | |
| --- | --- | --- | --- | --- | --- | --- | --- | --- | --- | --- | --- | --- | --- |
|  | No. | Writing | Drawing | Throwing | Scissors | Toothbrush | Chopsticks | Spoon | Brooming | Rubber | Opening boxes | LQ | Handedness |
| Intervention group | 1 | B | B | L | L | L | L | B | L | B | B | -33 | ABD |
|  | 2 | R | L | R | R | L | R | R | L | L | B | 9 | ABD |
|  | 3 | R | R | L | L | L | B | B | B | L | L | -23 | ABD |
|  | 4 | R | B | L | L | L | L | L | L | R | L | -45 | ABD |
|  | 5 | R | L | L | L | L | L | L | L | L | L | -80 | Left |
|  | 6 | R | B | L | L | L | L | L | B | L | B | -38 | ABD |
|  | 7 | B | L | L | B | L | B | L | L | B | L | -43 | ABD |
|  | 8 | R | R | L | L | L | R | L | L | L | L | -40 | ABD |
| Total | L | 0 | 3 | 7 | 6 | 8 | 4 | 5 | 6 | 5 | 5 |  |  |
|  | B | 2 | 3 | 0 | 1 | 0 | 2 | 2 | 2 | 2 | 3 |  |  |
|  | R | 6 | 2 | 1 | 1 | 0 | 2 | 1 | 0 | 1 | 0 |  |  |
| Control group | 9 | L | L | L | L | L | L | L | L | L | L | -100 | Left |
|  | 10 | L | L | L | L | L | L | L | L | L | L | -100 | Left |
|  | 11 | L | L | L | L | L | L | L | L | L | L | -100 | Left |
|  | 12 | L | L | L | L | L | L | L | L | L | L | -100 | Left |
|  | 13 | L | L | L | R | L | L | L | L | L | L | -80 | Left |
|  | 14 | L | B | L | L | L | L | L | L | L | L | -82 | Left |
|  | 15 | L | L | L | L | L | L | L | L | R | L | -80 | Left |
|  | 16 | L | L | L | L | L | L | L | L | L | L | -100 | Left |
|  | 17 | L | L | L | L | L | L | L | L | L | L | -100 | Left |
|  | 18 | L | L | L | L | L | L | B | L | L | L | -82 | Left |
|  | 19 | L | L | L | L | L | L | L | L | L | L | -100 | Left |
| Total | L | 11 | 10 | 11 | 10 | 11 | 11 | 10 | 11 | 10 | 11 |  |  |
|  | B | 0 | 1 | 0 | 0 | 0 | 0 | 1 | 0 | 0 | 0 |  |  |
|  | R | 0 | 0 | 0 | 1 | 0 | 0 | 0 | 0 | 1 | 0 |  |  |
| Laterality Quotient (LQ) = (L–R) / (L+R) ×100, L/R represent the total amount of left/right hand preference in daily activities respectively; LQ ≤ –50: Left-handed; LQ ≥ +50: Right-handed; -50＜LQ＜+50: Ambidextrous (ABD);  L: left; R: right; B: bilateral. | | | | | | | | | | | | | |
