# Supplementary material for: Changing handedness after nerve reconstruction in brachial plexus birth palsy
Source: Front Neurol. 2024 Jan 8;14:1284945. doi: 10.3389/fneur.2023.1284945 (PMC10800742; doi:10.3389/fneur.2023.1284945)
Supplement: Supplementary file 2 [file Table_2.doc]

| **Table S2. Semantic judgment response time and accuracy, semantic association activated voxel, LI and lateralization (n = 19)** | | | | | | | |
| --- | --- | --- | --- | --- | --- | --- | --- |
| No. | Semantic Judgment | |  | Semantic Association | | | |
|  | Reaction time（ms） | Accuracy（%） |  | Voxels in left hemisphere | Voxels in right hemisphere | LI | Lateralization |
| Intervention group | | | | | | | |
| 1 | 838.8 | 86.7 |  | 8922 | 14484 | -0.24 | Right |
| 2 | 863.4 | 93.3 |  | 8747 | 6486 | 0.15 | No |
| 3 | 862.4 | 86.7 |  | 1444 | 6819 | -0.65 | Right |
| 4 | 901.7 | 86.7 |  | 2530 | 2928 | -0.07 | No |
| 5 | 790.3 | 86.7 |  | 3619 | 6444 | -0.28 | Right |
| 6 | 849.3 | 86.7 |  | 11283 | 7873 | 0.18 | No |
| 7 | 829.5 | 100 |  | 12047 | 6533 | 0.29 | Left |
| 8 | 811.1 | 86.7 |  | 10327 | 7462 | 0.16 | No |
| Control group | | | | | | | |
| 9 | 866.5 | 86.7 |  | 3581 | 11879 | -0.54 | Right |
| 10 | 815.0 | 86.7 |  | 2462 | 7805 | -0.52 | Right |
| 11 | 877.1 | 86.7 |  | 2364 | 11634 | -0.66 | Right |
| 12 | 861.6 | 100 |  | 1467 | 11573 | -0.78 | Right |
| 13 | 812.3 | 86.7 |  | 1313 | 2037 | -0.22 | Right |
| 12 | 805.6 | 86.7 |  | 2697 | 6444 | -0.41 | Right |
| 15 | 844.7 | 100 |  | 1340 | 13146 | -0.82 | Right |
| 16 | 826.4 | 86.7 |  | 2496 | 9370 | -0.56 | Right |
| 17 | 844.5 | 93.3 |  | 2560 | 12781 | -0.67 | Right |
| 18 | 787.8 | 86.7 |  | 3724 | 10472 | -0.48 | Right |
| 19 | 838.9 | 86.7 |  | 4639 | 13538 | -0.49 | Right |
| LI: Lateralization Index (left-lateralized > 0.20, right-lateralized < -0.20, no lateralization -0.20 ~ 0.20). | | | | | | | |
|  | | | | | | | |
