# Supplementary material for: Changing handedness after nerve reconstruction in brachial plexus birth palsy
Source: Front Neurol. 2024 Jan 8;14:1284945. doi: 10.3389/fneur.2023.1284945 (PMC10800742; doi:10.3389/fneur.2023.1284945)
Supplement: Supplementary file 3 [file Table_3.doc]

| **Table S3. Handedness, dominant writing hand, writing speed,**  **language score and IQ (n = 19)** | | | | | |
| --- | --- | --- | --- | --- | --- |
| No. | Handedness | Writing hand | Speed（words/min） | Language score | IQ |
| Intervention group | | | | | |
| 1 | ABD | Right | 33.3 | 67 | 105 |
| 2* | ABD | Right | 44.2 | 68 | 100 |
| 3* | ABD | Right | 42.4 | 67 | 100 |
| 4* | ABD | Right | 54.3 | 71 | 105 |
| 5† | Left | Right | -- | 69 | 90 |
| 6 | ABD | Right | 19 | 67 | 90 |
| 7 | ABD | Right | 32 | 71 | 110 |
| 8 | ABD | Right | 32 | 67 | 105 |
| Control group | | | | | |
| 9 | Left | Left | 39.1 | 68 | 90 |
| 10* | Left | Left | 52.6 | 69 | 95 |
| 11* | Left | Left | 41.0 | 67 | 90 |
| 12* | Left | Left | 53.8 | 71 | 100 |
| 13 | Left | Left | 31.8 | 69 | 90 |
| 12* | Left | Left | 42.7 | 70 | 95 |
| 15* | Left | Left | 51.0 | 71 | 105 |
| 16* | Left | Left | 42.4 | 69 | 95 |
| 17* | Left | Left | 48.5 | 68 | 95 |
| 18* | Left | Left | 52.6 | 70 | 90 |
| 19* | Left | Left | 56.8 | 71 | 90 |
| †: The subject is left-handed, but right hand writing, thus writing speed was excluded;  *: Writing speed reaches normal;  ABD: Ambidextrous. | | | | | |
